# White matter hyperintensities in progranulin-associated frontotemporal dementia: A longitudinal GENFI study

**DOI:** 10.1016/j.nicl.2019.102077

**Published:** 2019-11-06

**Authors:** Carole H. Sudre, Martina Bocchetta, Carolin Heller, Rhian Convery, Mollie Neason, Katrina M. Moore, David M. Cash, David L. Thomas, Ione O.C. Woollacott, Martha Foiani, Amanda Heslegrave, Rachelle Shafei, Caroline Greaves, John van Swieten, Fermin Moreno, Raquel Sanchez-Valle, Barbara Borroni, Robert Laforce, Mario Masellis, Maria Carmela Tartaglia, Caroline Graff, Daniela Galimberti, James B. Rowe, Elizabeth Finger, Matthis Synofzik, Rik Vandenberghe, Alexandre de Mendonça, Fabrizio Tagliavini, Isabel Santana, Simon Ducharme, Chris Butler, Alex Gerhard, Johannes Levin, Adrian Danek, Giovanni B. Frisoni, Sandro Sorbi, Markus Otto, Henrik Zetterberg, Sebastien Ourselin, M. Jorge Cardoso, Jonathan D. Rohrer, GENFI

**Affiliations:** a School of Biomedical Engineering and Imaging Sciences, King's College London, UK; b Dementia Research Centre, Department of Neurodegenerative Disease, UCL Queen Square Institute of Neurology, London WC1N 3BG, UK; c Centre for Medical Image Computing, University College London, UK; d Department of Neurology, Erasmus Medical Centre, Rotterdam, Netherlands; e Cognitive Disorders Unit, Department of Neurology, Donostia University Hospital, San Sebastian, Gipuzkoa, Spain; f Alzheimer's disease and Other Cognitive Disorders Unit, Neurology Service, Hospital Clínic, Institut d'Investigacións Biomèdiques August Pi I Sunyer, University of Barcelona, Barcelona, Spain; g Centre for Neurodegenerative Disorders, Neurology Unit, Department of Clinical and Experimental Sciences, University of Brescia, Brescia, Italy; h Clinique Interdisciplinaire de Mémoire, Département des Sciences Neurologiques Université Laval Québec, Québec, Canada; i Sunnybrook Health Sciences Centre, Sunnybrook Research Institute, University of Toronto, Toronto, Canada; j Tanz Centre for Research in Neurodegenerative Diseases, University of Toronto, Toronto, Canada; k Department of Geriatric Medicine, Karolinska University Hospital-Huddinge, Stockholm, Sweden; l University of Milan, Centro Dino Ferrari, Milan, Italy; m Fondazione IRCCS Ca’ Granda, Ospedale Policlinico, Neurodegenerative Diseases Unit, Milan, Italy; n Department of Clinical Neurosciences, University of Cambridge, Cambridge, UK; o Department of Clinical Neurological Sciences, University of Western Ontario, London, Ontario Canada; p Department of Neurodegenerative Diseases, Hertie-Institute for Clinical Brain Research and Center of Neurology, University of Tübingen, Tübingen, Germany; q Laboratory for Cognitive Neurology, Department of Neurosciences, KU Leuven, Leuven, Belgium; r Faculty of Medicine, University of Lisbon, Lisbon, Portugal; s Fondazione Istituto di Ricovero e Cura a Carattere Scientifico Istituto Neurologica Carlo Besta, Milano, Italy; t Faculty of Medicine, University of Coimbra, Coimbra, Portugal; u Department of Psychiatry, McGill University Health Centre, McGill University, Montreal, Québec, Canada; v Department of Clinical Neurology, University of Oxford, Oxford, UK; w Faculty of Medical and Human Sciences, Institute of Brain, Behaviour and Mental Health, University of Manchester, Manchester, UK; x Department of Neurology, Ludwig-Maximilians-University, Munich, Germany; y Instituto di Recovero e Cura a Carattere Scientifico Istituto Centro San Giovanni di Dio Fatebenefratelli, Brescia, Italy; z Department of Neuroscience, Psychology, Drug Research, and Child Health, University of Florence, Florence, Italy; a1 Department of Neurology, University of Ulm, Ulm, Germany

**Keywords:** Frontotemporal dementia, White matter hyperintensities, Dementia, Progranulin, CSF, Cerebrospinal fluid, FTD, Frontotemporal dementia, GENFI, GENetic Frontotemporal dementia Initiative, GFAP, Glial Fibrillary Acidic Protein, GM, Grey Matter, GRN, Progranulin, MRI, Magnetic Resonance Imaging, WM, White Matter, WMH, White Matter Hyperintensity

## Abstract

Frontotemporal dementia (FTD) is a heterogeneous group of neurodegenerative disorders with both sporadic and genetic forms. Mutations in the progranulin gene (*GRN*) are a common cause of genetic FTD, causing either a behavioural presentation or, less commonly, language impairment. Presence on T2-weighted images of white matter hyperintensities (WMH) has been previously shown to be more commonly associated with *GRN* mutations rather than other forms of FTD. The aim of the current study was to investigate the longitudinal change in WMH and the associations of WMH burden with grey matter (GM) loss, markers of neurodegeneration and cognitive function in *GRN* mutation carriers.

336 participants in the Genetic FTD Initiative (GENFI) study were included in the analysis: 101 presymptomatic and 32 symptomatic *GRN* mutation carriers, as well as 203 mutation-negative controls. 39 presymptomatic and 12 symptomatic carriers, and 73 controls also had longitudinal data available. Participants underwent MR imaging acquisition including isotropic 1 mm T1-weighted and T2-weighted sequences. WMH were automatically segmented and locally subdivided to enable a more detailed representation of the pathology distribution. Log-transformed WMH volumes were investigated in terms of their global and regional associations with imaging measures (grey matter volumes), biomarker concentrations (plasma neurofilament light chain, NfL, and glial fibrillary acidic protein, GFAP), genetic status (*TMEM106B* risk genotype) and cognition (tests of executive function).

Analyses revealed that WMH load was higher in both symptomatic and presymptomatic groups compared with controls and this load increased over time. In particular, lesions were seen periventricularly in frontal and occipital lobes, progressing to medial layers over time. However, there was variability in the WMH load across *GRN* mutation carriers – in the symptomatic group 25.0% had none/mild load, 37.5% had medium and 37.5% had a severe load – a difference not fully explained by disease duration. GM atrophy was strongly associated with WMH load both globally and in separate lobes, and increased WMH burden in the frontal, periventricular and medial regions was associated with worse executive function. Furthermore, plasma NfL and to a lesser extent GFAP concentrations were seen to be associated with increased lesion burden. Lastly, the presence of the homozygous *TMEM106B* rs1990622 TT risk genotypic status was associated with an increased accrual of WMH per year.

In summary, WMH occur in *GRN* mutation carriers and accumulate over time, but are variable in their severity. They are associated with increased GM atrophy and executive dysfunction. Furthermore, their presence is associated with markers of WM damage (NfL) and astrocytosis (GFAP), whilst their accrual is modified by *TMEM106B* genetic status. WMH load may represent a target marker for trials of disease modifying therapies in individual patients but the variability across the *GRN* population would prevent use of such markers as a global outcome measure across all participants in a trial.

• White matter hyperintensities (WMH) accumulate over time in progranulin mutation carriers.
• WMH in GRN mutation carriers are associated with GM atrophy.
• WMH in GRN mutation carriers are associated with executive dysfunction.
• WMH load is variable across GRN mutation carriers.

## 1 Introduction

Frontotemporal dementia (FTD) is a neurodegenerative disorder with both familial and sporadic forms. Around a third of cases are genetic with mutations in three genes accounting for the majority of familial FTD: progranulin (*GRN*), microtubule-associated protein tau (*MAPT*) and chromosome 9 open reading frame 72 (*C9orf72)*. Magnetic resonance imaging (MRI) studies have shown progressive loss of grey matter (GM), particularly focused on the frontal and temporal lobes, in all three groups but the presence of white matter hyperintensities (WMH) is seen only in those with *GRN* mutations (Caroppo et al., 2014; Kelley et al., 2009; Sudre et al., 2017a).

Previous studies have shown that only a proportion of those with *GRN* mutations have high loads of WMH, with factors leading to the presence (or absence) of an increased burden still unclear. High levels of WMH are not purely related to disease severity as an increased load in presymptomatic *GRN* mutation carriers close to onset has also been reported (Sudre et al., 2017a). The underlying pathophysiological basis of WMH in *GRN* mutation carriers is also unknown, although prior studies have shown no association with vascular risk factors (Sudre et al., 2017a), and recent histopathological investigation has suggested that inflammation or astrocytosis may underlie the lesions (Woollacott et al., 2018).

The only factor that is known to modify phenotype in those with *GRN* mutations is a polymorphism in the *TMEM106B* gene (Nicholson and Rademakers, 2016). The risk genotype has been associated with an earlier age at onset (Cruchaga et al., 2011), decreased brain volumes (Harding et al., 2017), and impaired connectivity (Premi et al., 2014). However, no prior study has investigated its association with WMH.

In this study, we aimed to investigate the cross-sectional presence and the longitudinal change in WMH over time in *GRN*-associated FTD, hypothesizing that for a subgroup of cases, there would be an increase over time. We also aimed to examine the association between WMH burden and both GM atrophy and cognitive deficits in FTD, as well as the association with fluid markers of axonal damage (neurofilament light chain, NfL) and astrocytosis (glial fibrillary acidic protein, GFAP). Lastly, we investigated the association of WMH with the presence of the *TMEM106B* risk genotype.

## 2 Material and methods

### 2.1 Participants

Participants were recruited from the third data freeze of the Genetic FTD Initiative (GENFI), an international multicentre study of presymptomatic and symptomatic familial FTD (Rohrer et al., 2015). All participants undergo yearly clinical and cognitive assessment with MR imaging and fluid biomarker acquisition. All *GRN* mutation carriers and all controls (i.e. all mutation negative participants) with usable 3T volumetric T1- and T2-weighted MR scans were included in the study: 101 presymptomatic carriers, 32 symptomatic carriers and 203 controls were included (Table 1). For the longitudinal analysis, 124 participants (39 presymptomatic, 12 symptomatic carriers, and 73 controls) had follow-up imaging (70 with two scans, 28 with three, 21 with four and 5 with five).

**Table 1:** Baseline demographics, genetic status, biomarker concentrations and neuropsychological scores in controls, and both presymptomatic and symptomatic carriers. Significant differences are indicated by letters: a, between the symptomatic and control groups, and b, between the symptomatic and presymptomatic groups. Age, education, disease duration, neuropsychological tests (as z-scores), and NfL and GFAP concentrations are expressed as mean (standard deviation).

	Controls	Presymptomatic carriers	Symptomatic carriers
Number of participants	203	101	32
Female: male	117:86	65:36	18:14
*TMEM106B* genotype (CC:TC:TT)	6: 49: 36	1: 27: 11	1: 3: 6
Age (years)	46.5 (13.4)	45.5 (11.6)	64.4 (8.5)^a,b^
Education (years)	14.3 (3.3)	14.8 (3.6)	11.6 (3.6)^a,b^
Disease duration (years)	NA	NA	2.8 (2.1)
Trail Making Test part A (time)	−0.2 (0.7)	0.0 (0.7)	2.6 (3.2)^a^
Trail Making Test part B (time)	−0.2 (0.7)	−0.1 (0.7)	2.0 (2.5)^a^
WMS-R Digit Span Backwards (score)	0.0 (1.1)	−0.1 (1.1)	−1.6 (1.2)^a,b^
WAIS-R Digit Symbol test (score)	0.3 (1.0)	0.2 (1.0)	−2.1 (1.4)^a,b^
NfL concentration (pg/ml)	13.3 (17.7)	11.6 (9.4)	80.1 (42.6)^a,b^
GFAP concentration (pg/ml)	125.4 (64.4)	136.3 (69)	311.8 (170.6)^a,b^

There was no age difference between controls (mean 46.0, standard deviation 13.5) and presymptomatic (45.5, 11.6) groups (*p* = 0.51), but symptomatic *GRN* mutation carriers were significantly older than the other groups (64.3, 8.5). There were no differences in gender between the groups: 57.6% of the control population, 64.4% of the presymptomatic group and 56.3% of the symptomatic group were female.

### 2.2 MR acquisition

MR protocols had been harmonized at the start of the study and included a T1-weighted MPRAGE and a T2-weighted isotropic acquisition. Five scanners were used across different sites: 3 subjects were imaged on a GE Discovery MR750, 108 on a Philips Achieva, 51 on a Siemens Prisma, 72 on a Siemens Skyra and 102 on a Siemens Trio. Details of the acquisition protocol across the different scanners are reported as supplementary material (see Supplementary Table 1). The majority of the participants were scanned longitudinally on the same scanner but 32 were not scanned on the same scanner at all time points (24 controls, 9 presymptomatic mutation carriers and 1 symptomatic mutation carrier).

### 2.3 Neuropsychological testing

Participants underwent neuropsychological assessment (Rohrer et al., 2015). Prior studies have shown an association of WMH burden with tests of executive function and working memory (Dong et al., 2015; Kennedy and Raz, 2009; Prins et al., 2005), and so our analysis focused on a subset of tests from the GENFI battery: the Trail Making Test Parts A and B, WMS-R Digit Span Backwards, and WAIS-R Digit Symbol test. All scores were expressed as a z-score, with language-specific norms (Rohrer et al., 2015).

### 2.4 Biological sample acquisition and processing

Plasma samples were collected from 250 participants (152 controls, 75 presymptomatic and 23 symptomatic *GRN* mutation carriers) and centrifuged, aliquoted for plasma and stored at −80 °C (Rohrer et al., 2016). Samples were tested for NfL and GFAP using the Neurology 4-Plex A kit (102,153, Quanterix Corporation, Lexington, USA) on the SIMOA HD-1 Analyzer following manufacturer's instructions. To keep sample processing and plating consistent, participant samples were thawed at room temperature for two hours and subsequently centrifuged at 10,000 g for five minutes. 150 µl samples were aliquoted in duplicate in 96-well plates before testing. The lower limit of detection of the assay for the NfL and GFAP was 0.104 pg/ml and 0.221 pg/ml respectively. Measurements were carried out at a single site with the operator blinded to all clinical information, including genetic status.

The rs1990622 *TMEM106B* polymorphism status was available for 140 subjects: in total 53 had the TT (risk) genotype, 79 had the TC genotype and only 8 had the CC genotype.

### 2.5 Image analysis

The first step of the imaging analysis was to obtain the tissue segmentation and brain parcellation using an automated unified label fusion framework (Geodesic Information Flow - GIF) (Cardoso et al., 2015). The output of the label fusion algorithm provides subject-specific probability maps of anatomical tissues (GM WM, CSF, and others) that were used to initialise the WMH segmentation framework. Since the accuracy of the registration process at the core of the label fusion technique may be affected by the presence of WM lesions, and in turn affect the accuracy of the brain tissue segmentation, an iterative process was adopted to optimise the GM segmentation. This is notably important for the segmentation of subcortical structures such as caudate or putamen and overall measures of atrophy. Thus, to achieve a more accurate segmentation of GM regions, the two-step solution proposed by Valverde et al. (2014) in the context of multiple sclerosis was adopted; first the T1 weighted images were filled with normal appearing tissue (inpainting procedure) at the location of the detected lesions using the method described by Prados et al. (2016); second, once the T1 image was corrected, the label fusion algorithm was run again to provide the final GM segmentation.

In order to automatically segment the WMH acquired at multiple time points, the longitudinal extension of the framework presented by Sudre et al. (2017b) was used to limit intra-subject measurement noise. In the cross-sectional algorithm, the T2 image is rigidly registered (Modat et al., 2014) to the T1 image using the NiftyReg package (https://sourceforge.net/projects/niftyreg) and intensities are jointly modelled as a multivariate mixture of Gaussian distributions. This model allows for the simultaneous modelling of normal and unexpected observations (outliers), updating dynamically the number of required components to ensure the balance between fit to the data and model complexity. After convergence of the model, candidate WMH voxels are selected from the outlier components based on intensity and location constraints with respect to other tissues. The formed connected components are then automatically classified as lesions or artefacts thus preventing the presence of false positives. When using T2-weighted images, in order to avoid any ventricular segmentation, a 1 voxel border is excluded around the ventricles.

In the longitudinal extension of the described automated segmentation, an average image of all time points is first created from an iterative process that co-registers all time points to an average space, progressively increasing the allowed number of degrees of freedom while ensuring intensity matching between time points. The Gaussian mixture model is fitted on the obtained average image and finally used to constrain the segmentation at each individual time point.

In order to further characterize the location of WMH, the volume of the WM was subdivided using two schemes, following the method described in Sudre et al., 2018). The first scheme uses the parcellations from the label fusion technique to aggregate cortical regions into four lobes, as previously described (Rohrer et al., 2015); the WM is then divided into sub-regions according to the closest cortical lobe while the subcortical region is segmented independently. The second scheme uses the normalised distance between the ventricular surface and the cortical sheet to separate the WM into 4 equidistant layers. As the two mid layers (layers 2 and 3) are artificially divided without a clear biological division we merged them to form a single region, leaving three layers (peripheral, medial, and periventricular) for each of the lobes.

For both GM and WMH an asymmetry measure was calculated as the ratio of the difference between the left and right hemisphere and their sum.

### 2.6 Statistical analysis

Stata v.14 was used for all analyses. For all imaging derived dependent variables, age, gender, scanner type and total intracranial volume (TIV, measured using SPM12) were considered as covariates. Due to the skewness of the data, regional and local WMH volumes were log-transformed with an offset of 1 voxel to ensure the existence of the transformation.

Cross-sectional analysis used linear regression models with WMH burden at the latest time point as the dependent variable to investigate association with respect to participant clinical status (control, presymptomatic, symptomatic), *TMEM106B* genetic status, GM volume, NfL or GFAP concentration. For all models with imaging-derived dependent variables, age, gender, scanner type and TIV were included as covariates. For the analysis on *TMEM106B* genetic status, due to the very limited number of subjects with CC status, only subjects with TT or TC status were considered. When investigating the association with NfL and GFAP concentrations the time interval between biological sample and MR acquisition was further included as covariate. Apart from the investigation of the relationship with clinical status that explicitly distinguishes presymptomatic and symptomatic participants, all the other models were fitted for the whole subset of *GRN* mutation carriers and compared when necessary to the fit obtained for the control population.

In order to investigate cross-sectionally the relationship between neuropsychological tests and lesion volume in the *GRN* mutation carriers, the covariate-adjusted lesion volumes were used with respect to cognitive scores adjusted for age, gender, and years of education. Spearman correlation between corrected residuals was then used as a measure of the observed association.

As *GRN* mutation carriers have been commonly associated with asymmetrical GM atrophy (Rohrer et al., 2015) an analysis was performed to investigate whether GM asymmetry was associated with asymmetry of WMH using the Spearman correlation coefficient on the residuals after correction for age, gender, TIV and scanner type.

Longitudinally, a two-level linear mixed model to account for within subject scanner change was used with random slope and random intercept using the log transformed volume of WMH as a dependent variable. Similarly to the cross-sectional models, age, gender, scanner type and TIV were used as covariates.

Goodness of fit of the investigated models was assessed via test of gaussianity over the residuals using a Shapiro-Wilk test.

Due to the strong correlation between dependent variables, the results are presented without any correction for multiple comparisons following the rationale developed by Rothman (1990).

## 3 Results

### 3.1 Cross-sectional WMH burden (Fig. 1, Table 2)

Raw volumetric values are reported in Table 2. From the adjusted model, the overall total WMH burden was significantly higher in symptomatic participants compared to controls (excess of 48.2% [95% CI: 6.8, 105.7], *p* = 0.019) while there was a trend to a higher load in the presymptomatic group compared to controls (17.8% [−9.7, 39.7], *p* = 0.061). The symptomatic mutation carriers had a non-significantly higher overall burden compared to presymptomatic mutation carriers (25.8% [−10.4, 76.8], *p* = 0.184) (Table 2).

**Table 2:** Raw grey matter (GM) volumes and white matter hyperintensity (WMH) burden (total, by lobe [frontal, parietal, occipital and temporal] and by layer [periventricular, medial and peripheral]. GM volumes are presented as mean (standard deviation) while WMH volumes are reported as median [1st quartile; 3rd quartile]. Significant differences are indicated by letters: a, between the symptomatic and control groups, b, between the symptomatic and presymptomatic groups, c between the presymptomatic and control groups. All comparisons were performed with correction for age, gender, scanner and TIV. Log transformed volumes were used for the WMH.

		Controls	Presymptomatic carriers	Symptomatic carriers
GM (mL)	Frontal	177.0 (20.2)	178.9 (18.5)	141.5 (21.6)^a,b^
	Parietal	92.8 (10.5)	93.9 (10.1)	79.4 (9.4)^a,b^
	Occipital	72.7 (9.4)	73.3 (8.8)	68.1 (8.0)
	Temporal	119.9 (13.3)	120.0 (12.4)	105.8 (11.4)^a,b^
WMH (mm^3^)	Total	925.9 [576.0; 1375.2]	1037.3 [651.9; 1640.7]	1582.7 [925.0; 3541.2]^a^
	Frontal	225.8 [137.0; 402.9]	255.4 [146.5; 508.9]	988.0 [336.4; 1761.2]^a,b^
	Parietal	79.4 [33.1; 162.4]	84.6 [41.2; 196.9]^c^	152.9 [68.1; 365.7]
	Occipital	177.6 [109.1; 278.7]	208.0 [127.5; 310.2]^c^	334.7 [169.5; 534.4]^a^
	Temporal	235.4 [148.4; 354.8]	238.7 [161.8; 371.5]	160.8 [106.6; 334.4]^b^
	Periventricular	132.9 [74.1; 235.5]	143.4 [80.1; 277.6]	345.0 [185.2; 821.7]^a,b^
	Medial	302.0 [195.5; 499.6]	338.1 [219.1; 582.2]	725.4 [372.3; 2137.0]^a,b^
	Peripheral	432.9 [265.7; 686.4]	522.4 [278.4; 815.3]	431.3 [286.0; 835.8]^b^

In the lobar regions, the difference in burden was most noticeable in the frontal and occipital lobes. The symptomatic group had an excess WMH load of 116.3% ([35.4, 245.5], *p* = 0.001) and 59.2% ([13.8, 122.5], *p* = 0.006) respectively compared to controls. Symptomatic subjects also had a significantly higher load compared to the presymptomatic group in the frontal region 82.4% ([12.5, 195.0], *p* = 0.015). The presymptomatic group had a significantly higher load in both the occipital and parietal lobes compared to controls (21.6% [3.9, 42.3], *p* = 0.015; 36.9% [1.5, 84.5], *p* = 0.040 respectively).

With respect to the distance from the ventricles, the most periventricular region was significantly more affected in symptomatic subjects compared to both the presymptomatic group (excess of 79.9% [13.6, 184.8], *p* = 0.012) and controls (excess of 109% [34.9, 224.6], *p* = 0.001). WMH in this region in the presymptomatic group was non-significantly higher compared to controls (excess of 16.4% [−7.7, 46.8], *p* = 0.199). The medial region was significantly more affected in the symptomatic group compared to controls (91.1% [27.9, 185.6], *p* = 0.002) and in the symptomatic group compared to the presymptomatic group (65.2% [8.8, 150.8], *p* = 0.019), but this was not significant between the presymptomatic group and controls (15.7% [−4.8, 40.5], *p* = 0.142).

In order to further analyse the location of the lesions in the *GRN* mutation carriers with the highest WMH burden, the lesion maps were co-registered into MNI space, and the HARDI atlas of WM tractography (Zhang and Arfanakis, 2018) was used to determine which tracts were involved by ranking the number of voxels affected by WM lesions for each tract. Anteriorly the most affected tracts appeared to be linking the dorsal striatum with the superior and rostral frontal regions of the brain, along with the genu of the corpus callosum i.e. the fibre tract linking right and left frontal lobes (Fig. 2). Further back, the posterior parts of the superior longitudinal fasciculus and inferior fronto-occipital fasciculus joining the parietal and temporal lobes appeared to be the most affected.

**Fig. 1:**
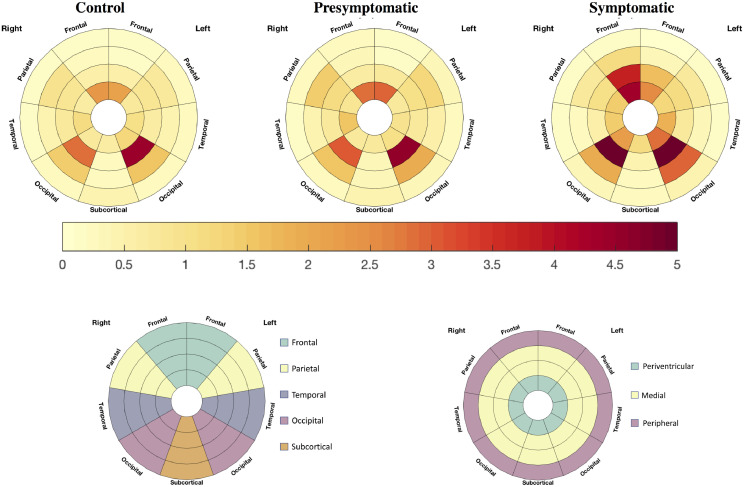
Top row: Marginal average of white matter hyperintensity (WMH) burden in the individual lobes and layers after correction for age, gender, scanner and TIV in controls, presymptomatic and symptomatic *GRN* mutation carriers. The bottom row shows a guide to the figures [left, lobar subdivision; right, layer subdivision]. The colour bar represents the average WMH load (increased = red, less = light yellow). (For interpretation of the references to color in this figure legend, the reader is referred to the web version of this article.)

**Fig. 2:**
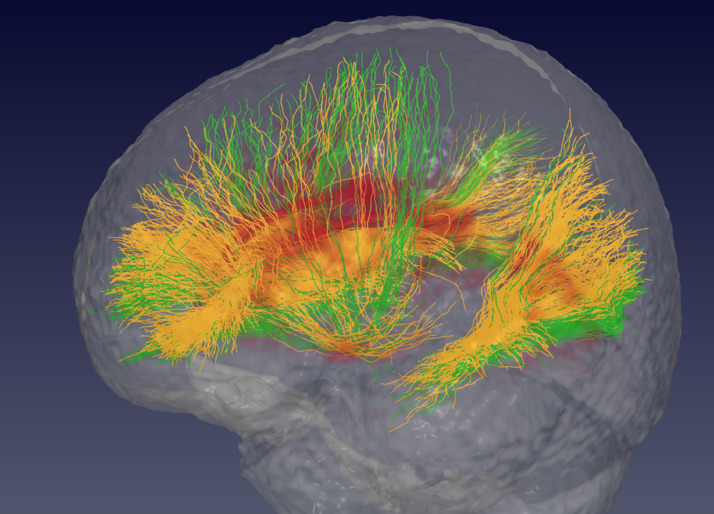
3D representation of the main tracts passing through the average white matter lesion maps of the *GRN* mutation carriers: in orange the tracts affected by the presence of lesions, and in green the tracts that do not go through lesions. The average lesion location is coloured in red. (For interpretation of the references to color in this figure legend, the reader is referred to the web version of this article.)

**Fig. 3:**
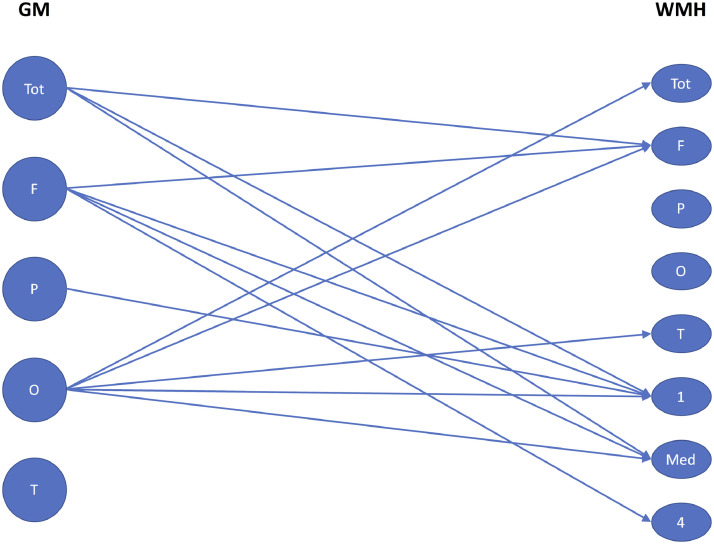
Significant associations between cross-sectional grey matter (GM) volume (Tot = total, *F* = frontal, *P* = parietal, *O* = occipital, *T* = temporal) and white matter hyperintensity (WMH) burden (Tot = total, *F* = frontal, *P* = parietal, *O* = occipital, *T* = temporal, 1 = periventricular layer, Med = medial layers, 4 = peripheral layer) within the *GRN* population. Significance is defined at a p-value threshold of 0.05 in the linear regression between GM volume and log transformed WMH volume after correction for age, gender, total intracranial volume and scanner type.

### 3.2 Longitudinal accumulation of wmh (Table 3)

The symptomatic group showed a trend to a greater longitudinal increase in WMH burden in the frontal lobe compared to controls (*p* = 0.075) but this was not significant when compared to presymptomatic subjects (*p* = 0.156). However there was a significant increase in the medial region compared to the presymptomatic group (*p* = 0.020) with a differential accrual of 15.5% [3.0, 28.9] per year (Table 3). No significant differences could be observed between the presymptomatic group and controls.

**Table 3:** Marginal mean and 95% confidence interval of longitudinal increase in white matter hyperintensities (WMH) per region (%/year). Significant differences are indicated by letters: a, between the symptomatic and presymptomatic groups.

	Controls	Presymptomatic carriers	Symptomatic carriers
Total	4.32 [−0.49; 9.35]	1.68 [−3.56; 7.21]	9.16 [−4.62; 24.93]
Frontal	6.27 [−0.17; 13.12]	10.38 [3.49; 17.72]	28.63 [5.28; 57.17]
Parietal	4.04 [−3.99; 12.73]	2.23 [−5.47; 10.56]	−1.32 [−20.01; 21.75]
Occipital	5.10 [−1.66; 12.31]	2.18 [−4.51; 9.34]	6.14 [−5.14; 18.77]
Temporal	7.37 [1.22; 13.9]	2.78 [−3.04; 8.95]	0.58 [−19.05; 24.98]
Periventricular	15.17 [4.99; 26.33]	12.58 [−0.15; 26.93]	42.76 [4.68; 94.71]
Medial	6.20 [0.56; 12.16]	0.79 [−6.02; 8.10]	16.86 [4.12; 31.16]^a^
Peripheral	4.79 [−0.23; 10.07]	4.91 [0.18; 9.87]	−5.44 [−25.64; 20.25]

### 3.3 Association with grey matter volume (Fig. 3)

A lower total GM volume was associated with a higher WMH burden in the frontal lobe and both periventricularly and medially for the *GRN* mutation carriers (combined presymptomatic and symptomatic). An overall decrease by 1 ml in the GM volume was associated with an increase of 0.83% ([95% CI=0.11,1.54], *p* = 0.024), 0.82% ([0.09, 1.55], *p* = 0.028), and 0.80% ([0.11, 1.48], *p* = 0.024) respectively. None of these associations were observed in control participants.

Lower volume of GM in the frontal lobe was associated with a larger volume of WMH in the same lobe for the *GRN* mutation carriers (*p* = 0.025), but no such association could be observed for the control group. Atrophy in all lobes except the temporal region was associated with larger WMH burden in the periventricular and medial regions. A 1 ml loss of GM volume in the frontal, parietal, and occipital lobes was respectively associated with an excess of 1.33% ([0.13, 2.54], *p* = 0.030), 2.54% ([−0.23, 5.32], *p* = 0.072), and 4.92% ([1.05, 8.84], *p* = 0.013) in the medial region. For the periventricular region, 1 ml of loss of GM volume in the frontal, parietal and occipital lobes were respectively associated with an excess of 1.35% ([0.13, 2.56], *p* = 0.030), 3.11% ([0.02, 6.20], *p* = 0.048), and 5.46% ([0.75, 10.17], *p* = 0.023).

Asymmetry measures of GM and WMH load were strongly associated in the frontal lobe in the symptomatic group (*r* = 0.28, *p* = 0.0006) but not in the other lobes or in the other groups.

Longitudinally, a lower baseline frontal GM volume was associated with an accelerated accrual in the medial region for the *GRN* mutation carriers (*p* = 0.0004, 0.1 ml less of frontal volume leading to 5.9% more of WMH per year). This relationship did not hold for the control group.

### 3.4 Association with cognition (Table 4)

A significant association between impaired cognition and WMH burden was found for the Digit Span Backwards in the frontal (*r* = −0.20, *p *= 0.02), periventricular (*r* = −0.23, *p* = 0.01), and medial (*r* = −0.23, *p* = 0.03) regions (Table 4) as well as the Digit Symbol test in the periventricular region (*r*=−0.18, *p* = 0.05, with a borderline association with the frontal region, *r*=−0.16, *p* = 0.08). Borderline associations were also seen in the periventricular and medial regions for the Trail Making Test Part A (*r* = 0.17, *p* = 0.06 for both).

**Table 4:** Spearman correlation coefficient between white matter hyperintensity (WMH) burden and cognitive scores after correction for age, gender, TIV and years of education (p value in parentheses). Significant correlations are shown in bold, and borderline associations (*p*<0.1) are in italics.

	Trail Making Test Part A	Trail Making Test Part B	WMS-R Digit Span Backwards	WAIS-R Digit Symbol Test
Total	0.13 (0.15)	0.00 (0.97)	*−0.16 (0.07)*	−0.06 (0.54)
Frontal	0.12 (0.17)	0.01 (0.95)	**−0.20 (0.02)**	*−0.16 (0.08)*
Parietal	0.02 (0.80)	−0.05 (0.57)	−0.12 (0.19)	−0.02 (0.87)
Occipital	0.06 (0.50)	−0.04 (0.68)	−0.10 (0.27)	0.01 (0.91)
Temporal	−0.04 (0.68)	−0.14 (0.12)	−0.01 (0.96)	0.13 (0.13)
Periventricular	*0.17 (0.06)*	0.03 (0.77)	**−0.23 (0.01)**	**−0.18 (0.05)**
Medial	*0.17 (0.06)*	0.03 (0.74)	**−0.20 (0.03)**	−0.10 (0.28)
Peripheral	−0.06 (0.51)	*−0.17 (0.06)*	−0.03 (0.77)	0.11 (0.22)

### 3.5 Association with fluid biomarkers

In the *GRN* mutation carriers there was a trend to an association between WMH and NfL concentration in the frontal lobe (WMH load excess of 0.63% [−0.14 1.41] per additional pg/ml of NfL, *p* = 0.107) and periventricularly (excess of 0.73% [−0.03, 1.51], *p* = 0.058) with significant associations in the medial region (excess of 0.67% [0.11 1.23], *p* = 0.020) and the occipital lobe (excess of 0.54% [0.13, 0.96], *p* = 0.011). There were no significant associations in the control population.

Longitudinally, a higher NfL concentration was associated with an increased WMH accrual in the medial layer (0.28%/year per additional pg/ml [0.15, 0.40], *p*<0.00001) and the occipital region (0.18 [0.04, 0.31], *p* = 0.008) for the *GRN* mutation carriers. A similar association was seen in the control population for the medial region with an additional accrual per year of 0.20% ([0.02 0.38], *p* = 0.029) but not for the occipital lobe (0.16% ([−0.14 0.46], *p* = 0.289).

The cross-sectional pattern seen with NfL was reproduced to a lesser extent when investigating the relationship between GFAP and WMH in the *GRN* mutation carriers. There was a trend to an association in the occipital lobe (a GFAP excess of 1pg/ml was associated with a larger WMH burden of 0.16% [−0.02 0.35], *p* = 0.078). This association was not significant for the periventricular layer (0.17% [−0.06, 0.41], *p* = 0.150). No associations were seen in the control population.

Longitudinally, in the *GRN* mutation carriers there was only a trend to an association between GFAP levels and WMH accrual in the occipital lobe (additional accrual of 0.03% [−0.006, 0.064] *p* = 0.100).

### 3.6 Association with TMEM106B polymorphism

Cross-sectionally, there appeared to be a weak relationship between *TMEM106B* genotype and WMH for the total burden in the *GRN* mutation carriers with higher WMH volume associated with the risk genotype: TT (+30.4% [−16.0; 102.4], *p* = 0.230). The association only reached significance for the parietal lobe WMH burden (+96.1% [0.55; 282.5], *p* = 0.048).

However, the longitudinal association between the risk *TMEM106B* genotype and increase in WMH was stronger. The TT group appeared to have a faster accumulation of total WMH (+8.2% per year overall [2.9; 13.6], *p* = 0.003) and in the medial region notably (+6.9% per year [2.1; 11.6], *p* = 0.005). A significant higher accrual of WMH in TT subjects was also observed in the temporal lobe (+13.1% per year [4.4; 21.7], *p* = 0.005). Such an association was not observed for the control group.

### 3.7 Grouping by WMH severity

Finally, we separated the presence of WMH in *GRN* mutation carriers into three groups (none/mild, moderate and severe loads) as is often done in other pathologies involving WMH such as multiple sclerosis, with a threshold at 1000 mm^3^ and 2500mm^3^ in a mean TIV of 1400ml (corresponding to an occupancy of 0.07% and 0.18% of the TIV) (Table 5). There was no significant difference in age between the none/mild and moderate groups in either the presymptomatic or symptomatic carriers although the severe group was older than both. However, in a separate analysis correcting for age, significant differences in WMH load were still found between the groups, suggesting that age was not the only factor driving group differences.

**Table 5:** Stratification of GRN population by white matter hyperintensity (WMH) burden severity into three groups: none or mild (0), moderate (1) and severe (2). Significant differences between groups are indicated in the last column. Results are shown as mean (standard deviation).

Group	None/Mild	Moderate	Severe	Significant differences
	0	1	2	
				
WMH (% of TIV)	0.0 (0.0)	1.1 (0.3)	4.2 (2.8)	
WMH (mm^3^)	644.8 (201.4)	1550.7 (430.2)	6118.2 (4473.7)	
Total number of carriers (Female:Male)	55 (37:18)	57 (34:23)	21 (9:12)	
Symptomatic carriers (number (%))	8 (25.0)	12 (37.5)	12 (37.5)	
Age (years)	59.0 (8.1)	62.1 (8.5)	70.2 (5.3)	0 vs 2, 1 vs 2
Disease duration (years)	1.6 (0.8)	3.8 (2.8)	2.5 (2.1)	0 vs 1
Presymptomatic carriers (number (%))	47 (46.5)	45 (44.5)	9 (8.9)	
Age (years)	43.8 (11.7)	45.9 (10.77)	52.6 (13.4)	0 vs 2, 1 vs 2
Grey matter (% of TIV)	35.2 (2.4)	34.9 (2.7)	31.7 (3.1)	0 vs 2, 1 vs 2
Trail Making Test Part A (time)	0.25 (1.15)	0.28 (1.74)	2.36 (3.34)	0 vs 2, 1 vs 2
Trail Making Test Part B (time)	0.19 (1.39)	0.25 (1.56)	1.15 (2.27)	0 vs 2, 1 vs 2
WMS-R Digit Span Backwards (score)	−0.08 (1.10)	−0.47 (1.31)	−1.30 (1.28)	0 vs 2
WAIS-R Digit Symbol test (score)	0.02 (1.21)	−0.32 (1.46)	−1.25 (1.73)	0 vs 2, 1 vs 2
NfL (pg/ml)	22.5 (32.5)	26.9 (38.1)	47.2 (39.9)	0 vs 2, 1 vs 2
GFAP (pg/ml)	152.6 (87.8)	174.2 (126.9)	270.2 (185.4)	0 vs 2, 1 vs 2

For the symptomatic cases, there was no significant difference in terms of disease duration between the individuals with most prominent WM damage (mean, standard deviation 2.5, 2.1 years) and the ones with none/mild WM (1.6, 0.8). GM volumes were significantly lower in the group with most severe WMH compared to both other groups (both when *GRN* mutation carriers were considered together, Table 5, and when split into symptomatic and presymptomatic groups, Supplementary Table 2). Performance on all four of the cognitive tests was significantly more impaired in the most severe group compared with the none/mild group (and on all but the Digit Span Backwards in the severe group compared to the moderate group) when the *GRN* mutation carriers were considered together (with significant group differences in the Trail Making Test Part A in presymptomatic carriers alone, and the Trail Making Test Part A and WAIS-R Digit Symbol test in symptomatic carriers alone, Supplementary Table 2). Plasma concentrations of NfL and GFAP were also significantly increased in the severe group compared to the other two groups.

## 4 Discussion

We have shown that WMH burden is increased in *GRN* mutation carriers compared with controls, and that this accumulation occurs particularly in the frontal and occipital regions, initially periventricularly and then extending out towards the cortex into the medial region. WMH burden increases over time in a subgroup of patients and is associated with GM volume loss as well as the presence of executive dysfunction. WMH burden is correlated with NfL concentration more strongly than GFAP concentration, and higher burden is associated with the *TMEM106B* rs1990622 risk genotype.

The cross-sectional finding of increased WMH in the symptomatic *GRN* group within periventricular and medial regions, particularly within the frontal and occipital lobes is consistent with prior studies (Caroppo et al., 2014; Kelley et al., 2009) including a previous smaller study in the GENFI cohort (Sudre et al., 2017a). However, this study extends those findings to show differences within the presymptomatic cohort, where significant differences were found in the parietal and occipital lobes. Despite these findings, there remains large variability within the *GRN* population – when classified into three groups of increasing severity, 25% of cases still have none or only mild WMH during the symptomatic phase, whilst 9% of the presymptomatic group already have severe WMH involvement.

A variable rate of longitudinal accrual of WMH was found in the *GRN* population, with the most significant increase in the medial region, suggesting a spread of WMH from initial periventricular regions outwards towards the cortex over time.

Forthcoming trials of disease-modifying therapy in *GRN* mutation carriers will require robust outcome measures. The presence of WMH cross-sectionally in only a subset of *GRN* mutation carriers and the variable accrual rate of WMH over time seems to preclude WMH volumes as being a global outcome measure across all participants (with the confidence intervals of calculated sample sizes being wide, and the upper limit extremely large). However, it may be possible to use WMH as markers within individual patients, and further work will be needed to investigate longitudinal changes over a longer period within the defined subset of *GRN* mutation carriers with WMH.

Frontal GM atrophy was found to be associated with frontal, periventricular and medial lesion load in the *GRN* mutation carriers but no such relationship could be found in the controls. Moreover, a longitudinal association between decreased baseline frontal GM volume and increased rate of WMH accrual in the medial region was seen. These findings are consistent with prior studies (Ameur et al., 2016; Caroppo et al., 2014; Kelley et al., 2009), and could be interpreted as Wallerian degeneration (McAleese et al., 2017, McAleese et al., 2015) involving a fronto-striatal circuit previously implicated in FTD (Looi et al., 2012). Notably, patients with *GRN* mutations have early striatal GM volume loss also (Rohrer et al., 2015). The clinical relevance of such findings may well be the known association of *GRN* mutations with parkinsonism (including corticobasal syndrome) (Möller et al., 2015; van Swieten and Heutink, 2008), and further investigation of the association between WMH and extrapyramidal symptoms will be important.

The clinical outcome of increased WMH burden appears to be worse executive function and slower information processing, with a significant association seen with performance on the Digit Span backwards and Digit Symbol test. This is consistent with studies in other conditions where WMH predominantly affect anterior areas of the brain (Kennedy and Raz, 2009). Prior neuroimaging studies of FTD have associated executive dysfunction with frontal cortical GM disease (Rosen et al., 2002) but the current study suggests that such cognitive deficits in *GRN* mutation carriers are likely to be due to a complex combination of GM and WM disease.

The association of NfL concentration with WMH burden is perhaps unsurprising as NfL is often felt to be a generic marker of axonal (and therefore WM) damage. However, NfL can be increased in FTD in the absence of WMH, and the increase of NfL in the *GRN* population is therefore likely to be a function of both WM tract disease not seen on T1 and T2 MR imaging and WMH. Future multimodal studies combining T1, T2 and diffusion tensor imaging will be helpful to investigate this further.

The trend towards an association between GFAP concentration and WM lesion load is consistent with a recent pathological study of WMH in a *GRN* mutation carrier that showed a strong association with the presence of astrogliosis (Woollacott et al., 2018). However there was only a weak relationship both cross-sectionally and longitudinally in our study and further work is required to better understand the role of GFAP and astrogliosis in the pathophysiology of WMH in *GRN* mutation carriers.

The risk genotype (TT) of the rs1990622 *TMEM106B* polymorphism was seen to be associated with an overall acceleration of WMH accrual over time in the *GRN* population but not in the control group. *TMEM106B* appears to regulate progranulin levels and disease penetrance in *GRN* mutation carriers (Finch et al., 2011), and the presence of the risk genotype is associated with lower GM volume (Harding et al., 2017) and impaired functional connectivity in the brain (Premi et al., 2017). This study adds to the knowledge about the role of *TMEM106B* in *GRN* mutation carriers and further work is needed to understand how the presence of the risk genotype leads to an increased accrual of WMH.

The underlying nature of the WMH in *GRN* mutation carriers has yet to be determined, although prior imaging and neuropathological work suggests that the lesions are not likely to be vascular (Sudre et al., 2017a; Woollacott et al., 2018) despite a relationship of progranulin with systemic metabolic disease (Nguyen et al., 2013), but instead are potentially inflammatory, with evidence of regional microglial dysfunction (Woollacott et al., 2018; Sakae et al., 2019). Recent studies suggest that lysosomal dysfunction within microglia is a key pathophysiological mechanism in *GRN* mutation carriers (Götzl et al., 2018), and that this is associated with *TMEM106B* function (Klein et al., 2017), hence providing a link to the findings in this study of a relationship between WMH and the *TMEM106B* risk genotype.

Apart from the fact that T2-weighted imaging may not be the optimal sequence of choice to segment WMH (including increased difficulty in segmentation at the ventricular border and the issue of jointly enlarged perivascular spaces and WMH), limitations of the study may include the relatively limited number of longitudinal cases available for analysis. However further data freezes within the GENFI study will allow larger longitudinal analyses to be performed in the future.

In order to further validate the hypothesis of Wallerian degeneration linking GM loss and WM lesions, the longitudinal evolution of diffusion tensor imaging metrics on the tracts impacted by lesions will be useful to investigate. Additionally, recent studies have highlighted the role of neuroinflammation and microglial activation in *GRN* mutation carriers, and particularly an association of abnormal, dystrophic microglia with WMH (Woollacott et al., 2018): it will therefore be important to correlate WMH burden with measures of inflammation such as CSF markers or microglial PET imaging in future studies.

## Funding

This work was funded by the UK Medical Research Council, the Italian Ministry of Health, and the Canadian Institutes of Health Research as part of a Centres of Excellence in Neurodegeneration grant (CoEN015), the Wellcome/EPSRC Centre for Medical Engineering [WT 203148/Z/16/Z], IMI2 grant AMYPAD [115952], the MSCA-ITN-Demo [721820], and the Wellcome Flagship Programme in High-Dimensional Neurology. The Dementia Research Centre is supported by Alzheimer's Research UK, Brain Research Trust, and The Wolfson Foundation. This work was supported by the NIHR Queen Square Dementia Biomedical Research Unit and the NIHR UCL/H Biomedical Research Centre. CHS is supported by an Alzheimer's Society Junior Fellowship (AS-JF-17-011). JDR is supported by an MRC Clinician Scientist Fellowship (MR/M008525/1) and has received funding from the NIHR Rare Disease Translational Research Collaboration (BRC149/NS/MH). KD is supported by an Alzheimer's Society PhD Studentship (AS-PhD-2015-005). JBR is supported by the Wellcome Trust (103838) and the NIHR Cambridge Biomedical Research Centre. MM is supported by the Canadian Institutes of Health Research and the Ontario Research Fund. RL is supported by Réseau de médecine génétique appliquée, Fonds de recherche du Québec—Santé (FRQS). FT is supported by the Italian Ministry of Health. DG is supported by the Fondazione Monzino and Italian Ministry of Health, Ricerca Corrente. SS is supported by Cassa di Risparmio di Firenze (CRF 2013/0199) and the Ministry of Health RF-2010-2319722. SO is supported by the Engineering and Physical Sciences Research Council (EP/H046410/1, EP/J020990/1, EP/K005278), the Medical Research Council (MR/J01107X/1), the EU-FP7 project VPH-DARE@IT (FP7-ICT-2011-9-601055), and the National Institute for Health Research University College London Hospitals Biomedical Research Centre (NIHR BRC UCLH/UCL High Impact Initiative BW.mn.BRC10269). JvS is supported by The Netherlands Organisation for Health Research and Development Memorable grant (733050103) and Netherlands Alzheimer Foundation Memorable grant (733050103).

## Declaration of Competing Interest

The authors declare no competing interests.

## Appendix Supplementary materials

Image, application 1
