# Supplementary material for: White matter hyperintensities in progranulin-associated frontotemporal dementia: A longitudinal GENFI study
Source: Neuroimage Clin. 2019 Nov 6;24:102077. doi: 10.1016/j.nicl.2019.102077 (PMC6911860; doi:10.1016/j.nicl.2019.102077)
Supplement: Supplementary file 1 [file mmc1.docx]

**Supplementary Table 1**

**Details of MR sequence acquisition parameters for the T1- and T2-weighted imaging. Similar parameters were used longitudinally. Numbers are given for the cross-sectional analysis**

| **Modality** | **Scanner** | **Number**  **CTR/PS/S** | **Duration** | **Voxel/matrix size** | **Key parameters** |
| --- | --- | --- | --- | --- | --- |
| 3D T1 | Siemens Prisma | 34/12/5 | 5min 6s | 1.1x1.1x1.1mm^3^  256x256x208 | MPRAGE sagittal, 10% phase oversampling, iPAT=2, TI/TR=850/2000ms; flip angle 8° |
|  | Siemens Skyra | 44/21/7 | 8min 32s | 1.1x1.1x1.1mm^3^  256x256x208 | MPRAGE sagittal, iPAT=off, TI/TR=850/2000ms; flip angle 8° |
|  | Siemens Trio | 55/36/11 | 5min 6s | 1.1x1.1x1.1mm^3^  256x256x208 | MPRAGE sagittal, 10% phase oversampling, iPAT=2, TI/TR=850/2000ms; flip angle 8° |
|  | GE Discovery | 3/0/0 | 5min 6s | 1.1x1.1x1.1mm^3^  256x256x208 | IR-SPGR sagittal, ASSET=2, TI=400ms; inner loop TR=6.6ms, flip angle 8° |
|  | Philips | 67/32/9 | 4min 43s | 1.1x1.1x1.1mm^3^  256x256x208 | MPRAGE sagittal, SENSE=2, TI/TR=933/2200ms; flip angle 8° |
| 3D T2 | Siemens Prisma | 34/12/5 | 4min 43s | 1.1x1.1x1.1mm^3^  256x256x176 | Long echo train FSE, sagittal iPAT=2, TE_eff_/TR=105/3200ms, water-selective excitation |
|  | Siemens Skyra | 44/21/7 | 4min 46s | 1.1x1.1x1.1mm^3^  256x256x176 | Long echo train FSE, sagittal iPAT=2, TE_eff_/TR=105/3200ms |
|  | Siemens Trio | 55/36/11 | 4min 43s | 1.1x1.1x1.1mm^3^  256x256x176 | Long echo train FSE, sagittal iPAT=2, TE_eff_/TR=105/3200ms, water-selective excitation |
|  | GE Discovery | 3/0/0 | 4min 43s | 1.1x1.1x1.1mm^3^  256x256x208 | Long echo train FSE, sagittal ASSET=2, TE_eff_/TR=78.6/3200ms |
|  | Philips | 67/32/9 | 4min 37s | 1.1x1.1x1.1mm^3^  256x256x176 | Long echo train FSE, sagittal SENSE=2, TE_eff_/TR=103/2200ms |

**Supplementary Table 2**

**Volumetric GM volumes and cognition z-scores by WMH load severity category separating presymptomatic and symptomatic subjects**

| Group | | None/Mild | Moderate | Severe | Comparison |
| --- | --- | --- | --- | --- | --- |
|  |  | 0 | 1 | 2 |  |
| Presymptomatic | GM | 0.36 (0.01) | 0.36 (0.02) | 0.34 (0.03) | 0 vs 2, 1 vs 2 |
|  | Trail Making Test part A | 0.0 (0.6) | -0.2 (0.6) | 0.2 (1.2) | 1 vs 2 |
|  | Trail Making Test part B | -0.1 (0.9) | -0.2 (0.6) | -0.2 (0.4) |  |
|  | WAIS-R Span Backwards | 0.1 (1.0) | -0.1 (1.1) | -0.6 (1.2) |  |
|  | WAIS-R Digit Symbol test | 0.3 (0.9) | 0.1 (1.0) | 0.2 (0.8) |  |
| Symptomatic | GM | 0.30 (0.02) | 0.31 (0.03) | 0.30 (0.02) | 1 vs 2 |
|  | Trail Making Test part A | 1.3 (2.2) | 2.0 (3.1) | 3.9 (3.6) | 0 vs 2 |
|  | Trail Making Test part B | 1.7 (2.3) | 1.8 (2.8) | 2.2 (2.6) |  |
|  | WAIS-R Span Backwards | -0.9 (1.3) | -1.8 (1.3) | -1.9 (1.1) |  |
|  | WAIS-R Digit Symbol test | -1.6 (1.3) | -2.0 (1.6) | -2.4 (1.3) | 0 vs 2 |
